# Indocyanine green fluorescence during total thyroidectomy: utility for predicting hypoparathyroidism and safety assessment

**DOI:** 10.1007/s00423-026-04149-5

**Published:** 2026-07-27

**Authors:** Rasmus Reinke, Stefano Londero, Martin Almquist, Lars Rejnmark, Lars Rolighed

**Affiliations:** 1 Department of Otorhinolaryngology, Head and Neck Surgery, Aarhus University Hospital, Aarhus, Denmark; 2 Department of Surgery, Lund University Hospital, Lund, Sweden; 3 Department of Endocrinology and Internal Medicine, Aarhus University Hospital, Aarhus, Denmark

**Keywords:** Fluorescence, ICG, Hypoparathyroidism, Indocyanine Green, Total Thyroidectomy

## Abstract

**Purpose:**

Parathyroid fluorescence with indocyanine green (ICG) might prevent and predict postoperative hypoparathyroidism (hypoPT) after total thyroidectomy (TT). We aimed to evaluate parathyroid gland vascularization with ICG during TT. Further, we aimed to evaluate safety of ICG fluorescence and to correlate changes in PTH with postoperative hypoPT after TT.

**Methods:**

From May 2021 to June 2023, consecutive patients underwent TT and parathyroid fluorescence with ICG. Each visible parathyroid gland was scored 0, 1 or 2 depending on fluorescence intensity. Patients with at least one gland score of 2 were compared to those without. We measured PTH before and after TT, changes in calcium, creatinine and alanine transaminase (ALAT), postoperative alfacalcidol requirements, and adverse events of ICG use.

**Results:**

A total of 124 patients were included. Patients with at least one gland scored 2 (*n* = 108) had a reduced need for alfacalcidol at discharge compared with those without (8% vs. 38%, *p* = 0.004), although hypoPT was not fully excluded. Six patients required alfacalcidol after 12 months and were classified as having permanent hypoPT; all had an intraoperative PTH decrease > 83%. Creatinine levels were higher in patients with permanent hypoPT compared to others (*p* = 0.01). No increases in ALAT or ICG-related adverse events were observed.

**Conclusions:**

ICG-based parathyroid fluorescence during TT was well tolerated in this cohort and predicted immediate postoperative hypoPT. However, a fluorescence score of 2 did not exclude hypoPT development. The grading system is limited by subjectivity. No permanent hypoPT occurred in patients with a PTH decrease ≤ 83% after TT.

## Introduction

Total thyroidectomy (TT) is associated with a high risk of postoperative hypoparathyroidism [1, 2] (hypoPT). After surgery, hypoPT may develop due to damage of the glands, unintentional resection, or damage of the blood vessels supplying the parathyroid glands. The complication leads to insufficient plasma levels of parathyroid hormone (PTH). Patients with hypoPT risk multiple long-term complications including chronic kidney disease (CKD) [3–7] and reduced quality of life (QoL) [8–10].

To help reduce the risk of postoperative hypoPT, studies on parathyroid vascular supply have been performed [11]. With intravenous injection of indocyanine green (ICG) and specific cameras to detect fluorescence [12], the uptake and fluorescence of ICG in the parathyroid gland can be quantified and evaluated before, during, and after a thyroid procedure. Initial experience using ICG has demonstrated that one well-perfused parathyroid gland was sufficient to avoid permanent hypoPT[13]. Without ICG uptake, the risk of hypoPT seems markedly increased. Accordingly, the use of ICG to evaluate parathyroid function may be an important intraoperative tool during thyroidectomy.

Initially, ICG was developed as a tool for detecting macular degeneration [12] and ICG angiography has since found diverse applications in the medical field. Notably, it has been employed for identifying sentinel lymph nodes[14], and investigating hepatic function [15], as the ICG is primarily absorbed from the bloodstream by the hepatic parenchymal cells and then completely excreted into the bile [12]. However, despite the long-term use of ICG in the clinic, data on its safety remains sparse in the literature.

We aimed to perform an immediate evaluation of parathyroid glands during the operation and to measure operative changes in PTH levels. With a short half-life, the prediction of postoperative hypoPT can be estimated with measurements of changes in plasma PTH [16–18]. With four parathyroid glands, assuming equal hormone production between all glands, a PTH decrease > 75% may be associated with an increased risk of hypoPT [13]. As a secondary outcome, we aimed to evaluate the safety of ICG fluorescence by recording adverse events and measuring biochemical markers of liver and kidney function. Finally, long-term outcomes regarding hypoPT were recorded.

## Materials and methods

### Procedures

**Fig. 1:**
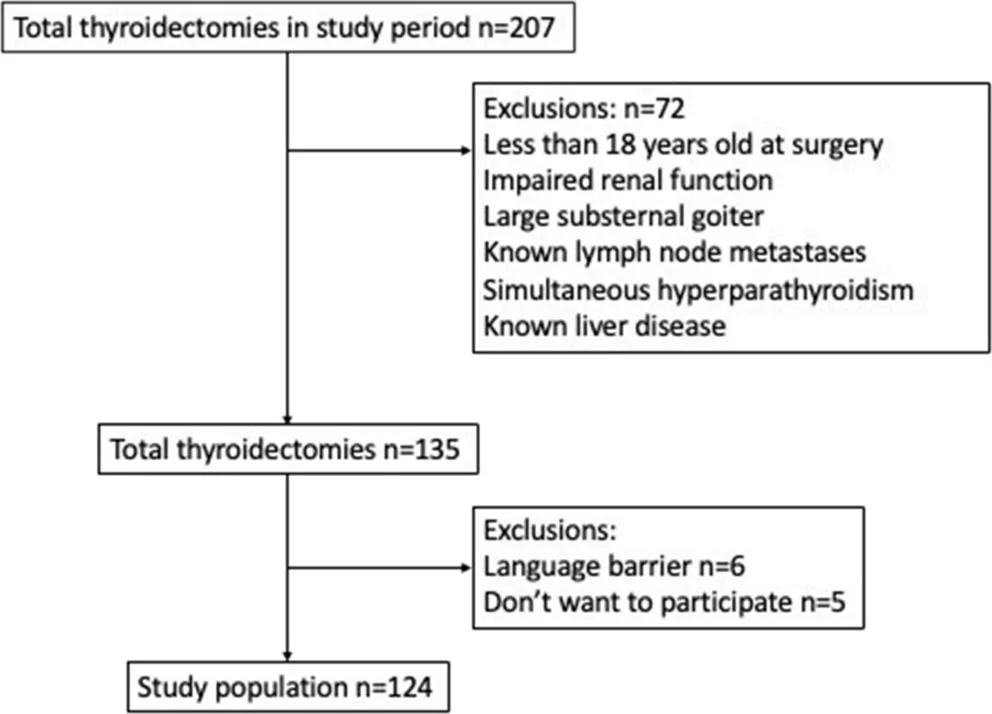
Flowchart for inclusion and exclusion of total thyroidectomy patients

This was a prospective nonrandomized cohort study of TT patients operated from May 2021 to June 2023 at a single surgical center. The inclusion criteria were patients aged 18 years or older with a clinical indication for TT during the study period. Exclusion criteria were impaired renal function (eGFR < 30 ml/min/1.73m^2^), large substernal goiter, thyroid cancer with known lymph node metastases, simultaneous hyperparathyroidism, or known liver disease (Fig. 1).

Before surgery, we measured plasma levels of parathyroid hormone (PTH), ionized calcium (Ca^2+^), alanine aminotransferase (ALAT), and creatinine in all patients. We measured PTH before incision and immediately after wound closure in each operation. Only two surgeons performed operations. Each visible parathyroid gland was scored independently by the operating surgeon at the time of the procedure; scores were not reviewed or discussed between surgeons. This approach maintains prospective independence of scoring but means formal inter-rater reliability could not be assessed, which is acknowledged as a limitation. At postoperative day 1 (POD1), we measured PTH and Ca^2+^. We repeated measurements of PTH, creatinine, ALAT, and Ca^2+^ after 2, 6, and 12 months. The use of active vitamin D analogs (alfacalcidol) was recorded in the patient records to determine hypoPT status and track the duration of the condition.

### Equipment

**Fig. 2:**
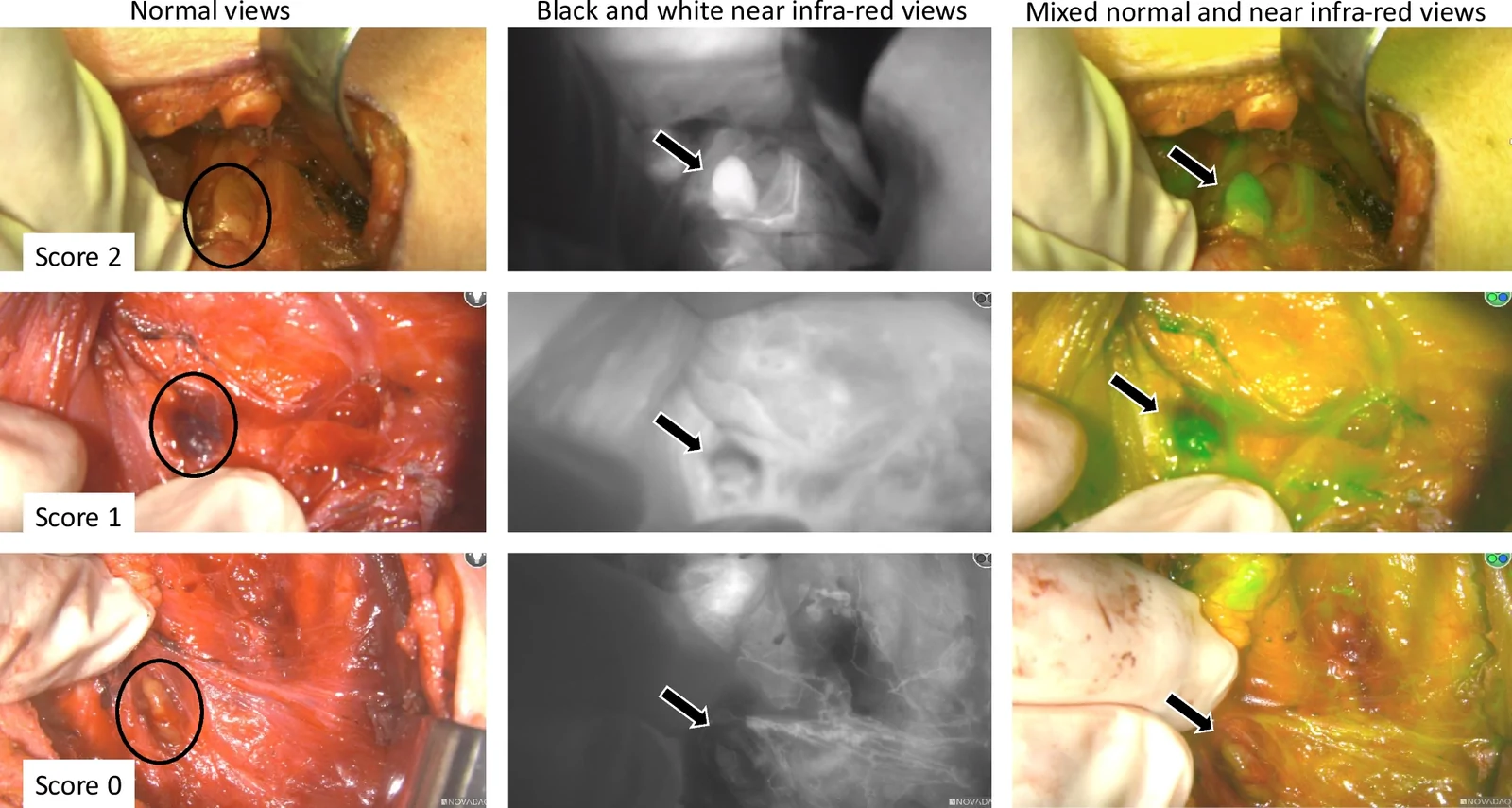
Grading of 3 representative parathyroid glands. Showing high uptake (Score 2), intermittent uptake (Score 1), or zero uptake of Indocyanine green (ICG) (Score 0). Three different camera modes are available in normal view, black and white near infra-red, or a mixture of normal and near infra-red. Circles surround parathyroid glands, and arrows indicate the glands with or without uptake of ICG

A SPY-PHI system from Stryker was used. The camera amplifies the fluorescence from a simultaneous infusion of ICG [12]. The fluorescent image is combined with the image from a normal high-definition camera as an on-lay image. The parathyroid glands are identified and preserved after open dissection on one side of the thyroid gland. Before the procedure continued on the contralateral side, the ICG injection was given and glands were inspected with the SPY-PHI camera. The normal dose for each side was 1 ml ICG (2.5 mg/ml). If the patient’s weight was above 100 kg the dose was increased to 1.5 ml. Perfusion of the parathyroid gland(s) on the first side was evaluated. This method makes it possible to evaluate the perfusion of the first two parathyroid glands before operating on the contralateral side. The intraoperative perfusion was evaluated using a score-based system ranging from 0–2 after the thyroid resection, previously used by Vidal et al. [12]. A score = 0; no perfusion of the gland, and therefore no fluorescent light emission from the parathyroid gland. A score = 1; detectable light was emitting from the gland suggesting that the gland is partially vascularized. A score = 2; optimal light emission signifying vital blood perfusion to the gland (Fig. 2). All intraoperative details from fluorescence were prospectively recorded in a RedCap database with each visible gland score noted.

### Definitions

Hypocalcemia was defined as Ca^2+^ levels below the lower limit of the reference interval (< 1.18 mmol/L). HypoPT was defined as a condition with hypocalcemia and levels of PTH insufficient to normalize Ca^2+^, necessitating treatment with alfacalcidol. Accordingly, we evaluated the need for treatment with active vitamin D (alfacalcidol) at POD1 and after 2, 6, and 12 months. In accordance with Danish national guidelines, all patients were admitted overnight following surgery. PTH and ionized calcium were measured at POD1. In patients with PTH values below the threshold, biochemistry was monitored and treated by endocrinologists, with the aim of gradually reduce supplementation as parameters normalized. Long-term treatment status was verified using the Danish national prescription database, which enabled reliable ascertainment of whether and when alfacalcidol treatment was discontinued.

### Statistics

Statistical analysis was performed using StataCorp. 2023. *Stata Statistical Software: Release 18*. College Station, TX: StataCorp LLC. Data was tested for distribution; continuous and normally distributed variables were expressed as mean with 95% confidence interval (95% CI) and compared using Student’s *t*-test either paired or unpaired depending on the distribution of the sample. Non-normally distributed variables were expressed by a median with interquartile range (IQR) and compared using the Mann–Whitney *U* test. Dichotomous variables were compared using the *χ*^2^ test. Lastly, empirical cutoff estimation was conducted using receiver operating characteristic (ROC) curve analysis, along with the Liu method, to calculate the optimal cutoff for predicting hypoPT based on the percentage decrease in PTH. For each patient, the percentage decrease in PTH was calculated. To assess the impact of fluorescence scores on predicting immediate hypoPT, we compared patients with at least one gland score of 2 to those without a single gland score of 2.

Safety was evaluated by recording adverse events and measuring ALAT and creatinine levels before surgery and at 2-, 6- and 12-months post-TT. Creatinine levels were further analyzed to compare patients with and without hypoPT. Statistical significance was set at a two-tailed *p*-value < 0.05. Exact binomial 95% confidence intervals were calculated for the sensitivity and specificity of the ROC-derived PTH cut-off. A formal a priori power calculation was performed before study initiation. Based on an estimated 30% of TT patients having insufficient ICG fluorescence and a background risk of permanent hypoPT of approximately 15% [19] (expected predominantly in the low-fluorescence group), 20 patients per group were required to achieve 80% power at a two-sided significance level of 5%. With approximately 80 thyroidectomies performed annually at our centre, we estimated full inclusion and 12-month follow-up could be completed within a 3-year period.

### Approvals

The project was approved by the Regional Ethics Committee with case nr.: **1–10–72–28-21**. All patients signed written agreements for voluntary participation in the study. No Research Resource Identifiers are applicable to this study.

## Results

### Patients

We included 124 patients in the study period (Fig. 1) with a mean age of 51 (19–89), of which (77%) were female, nearly half the patients were operated for thyrotoxicosis, whereas 36% and 15% had goiter and cancer as surgical indications, respectively. The median (IQR) thyroid gland weight was 66 (41–136) g and the median bleeding volume was 50 ml (20–50 ml), Table 1. The number of parathyroid glands identified was 4 in 49 patients, 3 in 55 patients, 2 in 19 patients, and 1 in a single patient.

**Table 1:** Descriptive summary of total thyroidectomy patients

Characteristics		Data
All	N (%)	124 (100)
Age	Mean (Range)	51 (19—89)
Female gender	N (%)	96 (77)
BMI (kg/m^2^)	Mean (Range)	28.2 (14.1—51.8)
Etiology	Goiter, N (%)	44 (36)
	Thyrotoxicosis, N (%)	61 (49)
	Cancer, N (%)	19 (15)
Thyroid weight (g)	Median (IQR)	66 (41;136)
Operative bleeding (ml)	Median (IQR)	50 (20;50)

### Intraoperative fluorescence

In 108 patients (87%) at least one parathyroid gland had a score of 2, while 16 (13%) did not have any visible glands with a score of 2 (Table 2). For those with a gland score of 2, the mean PTH decreased by 56% (95% CI: 52–61%) compared with a mean 71% decrease (95% CI: 58–83%) in patients without a score of 2 (*p* = 0.027). At POD1, both PTH and Ca^2+^ levels were higher in the group with a gland score of 2 although p-values were 0.06 and 0.14, respectively (Table 2). No significant difference was observed between the two groups regarding PTH or Ca^2+^ levels after 2 months. At discharge, patients without a score of 2 required alfacalcidol in 38% of cases, compared with 8% of patients with a score of 2 (*p* = 0.001). A similar pattern was seen at 2 and 6 months. However, after 12 months, no significant difference was found between patients with a gland score of 2 and those without (Table 2). There was no difference in other risk factors of hypoPT including etiology, bleeding, and thyroid weight (Table 2).

**Table 2:** Comparison of patients with and without at least one parathyroid gland with an indocyanine green score of 2

	Gland score 2	No gland score 2	*P*-value
Number of patients, N (%)	108 (87)	16 (13)	
PTH change (%), Mean (95% CI)	56 (52–61)	71 (58–83)	**0.027**
PTH POD1 (pmol/L), Mean (95% CI) *n *= 124	3.5 (3.0–3.9)	2.4 (1.3–3.5)	0.06
Ca^2+^ POD1 (mmol/L), Mean (95% CI)	1.20 (1.19–1.21)	1.17 (1.13–1.21)	0.14
Etiology			
Goiter, N (%)	37 (34%)	7 (44%)	
Graves, N (%)	53 (49%)	8 (50%)	
Cancer, N (%)	18 (17%)	1 (6%)	
Thyroid weight (g), Median (IQR)	66 (41;136)	64 (37;124)	0.94
Operative bleeding (ml), Median (IQR)	50 (20;63)	50 (20;50)	0.88
PTH 2 months (pmol/L), Mean (95% CI) *n* = 103	5.3 (4.6–6.0)	5.0 (3.9–6.0)	0.74
PTH 6 months (pmol/L), Mean (95% CI) *n* = 81	4.8 (4.2–5.3)	6.2 (3.6–8.9)	0.16
PTH 12 months (pmol/L), Mean (95% CI) *n* = 79	5.8 (4.9–6.6)	6.1 (3.6–8.6)	0.74
Ca^2+^ 2 months (mmol/L), Mean (95% CI) *n* = 113	1.24 (1.23–1.26)	1.23 (1.20–1.26)	0.63
Alfacalcidol at discharge, N (%)	9 (8)	6 (38)	**0.001**
Alfacalcidol at 2 months, N (%)	7 (6)	4 (25)	**0.015**
Alfacalcidol at 6 months, N (%)	4 (4)	4 (25)	**0.001**
Alfacalcidol at 12 months, N (%)	4 (4)	2 (12.5)	0.13

### Safety of ICG fluorescence

We recorded no allergic reactions during or after the injection of ICG. Despite the excretion of ICG from the liver, no increase was measured in ALAT values over time. However incidentally, we identified a small decrease in ALAT within the normal range 2 months after TT. Preoperative ALAT values was U/L (95% CI) 27.6 (24.7–30.54 *n* = 122) and after 2 months 24.9 (22.2–27.6 *n* = 116) *p* = 0.007.

The kidneys do not assist in ICG elimination; however, creatinine measurements were included in safety measurements. We measured a small but significant increase in creatinine in all patients. The mean increase in creatinine level (μmol/L, 95% CI) was 6.5 (4.4–8.6, *n* = 108) after 12 months. For patients in treatment for hypoPT after 12 months the mean increase was 17.5 (0.6–34.4, *n* = 6), significantly higher than those without hypoPT, 5.9 (3.8–7.9, *n* = 102) *p* = 0.01.

### Hypoparathyroidism

The frequency of treatment with alfacalcidol was 12% at discharge, 9% after two months, 6.4% after six months, and 4.8% after twelve months (6 patients, Table 3). After twelve months, calcium and PTH were within reference range in 4 of the 6 hypoPT patients. Two patients (1.6%) still presented with unmeasurable PTH levels. Treatment varied from 0.5–3 μg alfacalcidol, calcium (500–800 mg), and vitamin D (19–38 μg). No significant difference was found in ALAT values between hypoPT and non-hypoPT patients.

**Table 3:** Comparison of patients with PTH decrease below or above 75% during total thyroidectomy

Decrease in PTH	< 75%	> 75%	*P*-value
Number of patients (%)	87 (71)	37 (29)	
PTH change (%), Mean (95% CI)	47 (43–51)	86 (84–88)	** < 0.001**
ICG score = 2, N (%)	79 (89)	29 (81)	0.16
PTH POD1 (pmol/L). Mean (95% CI)	4 (3.6–4.5)	1.7 (1.2–2.2)	** < 0.001**
Ca^2+^ POD1 (mmol/L), Mean (95% CI)	1.22 (1.20–1.23)	1.14 (1.12–1.17)	** < 0.001**
Etiology			
Goiter, N (%)	31 (35%)	13 (36%)	
Graves, N (%)	43 (49%)	18 (50%)	
Cancer, N (%)	14 (16%)	5 (14%)	
Thyroid weight (g), Median (IQR)	58 (37; 122)	76 (49; 144)	0.17
Operative bleeding (ml), Median (IQR)	30 (20; 50)	50 (30; 100)	0.054
PTH 2 Months (pmol/L), Mean (95% CI) (*n* = 104)	5.8 (5.0–6.6)	3.9 (3.2–4.7)	**0.004**
PTH 6 Months (pmol/L), Mean (95% CI) (*n* = 81)	5.7 (4.8–6.5)	3.6 (2.8–4–8)	**0.003**
PTH 12 Months (pmol/L), Mean (95% CI) (*n* = 79)	6.3 (5.2–7.4)	4.9 (3.9–5.8)	0.08
Ca^2+^ 2 Months (mmol/L), Mean (95% CI) (*n* = 109)	1.24 (1.23–1.25)	1.23 (1.21–1.24)	0.31
Alfacalcidol at discharge, N (%)	0 (0)	15 (41.7)	** < 0.001**
Alfacalcidol at 2 months, N (%)	0 (0)	11 (29.7)	** < 0.001**
Alfacalcidol at 6 months, N (%)	0 (0)	8 (21.6)	** < 0.001**
Alfacalcidol at 12 months, N (%)	0 (0)	6 (16.2)	** < 0.001**

### Decrease in PTH

As a secondary outcome, we evaluated whether an intraoperative PTH decrease below 75% could exclude postoperative hypoPT. Data are summarized in Table 3. In 87 patients (71%) with a PTH decrease less than 75%, none were prescribed alfacalcidol at discharge or at 2, 6, or 12 months. Accordingly, a PTH decrease below this level seems to exclude permanent hypoPT. Notably, the 75% threshold is used here as a clinically established reference point from prior literature; the ROC-derived optimal cut-off of 84% described below reflects the empirical threshold in our specific cohort for predicting permanent hypoPT and should be interpreted in that context.

On the first day after surgery (POD1), patients whose PTH dropped by more than 75% during the operation had lower mean PTH levels (1.7 vs. 4 pmol/L, *p* < 0.001) and lower mean calcium levels (1.14 vs. 1.22 mmol/L, *p* < 0.001) compared with those with smaller PTH decreases. These differences were still significant at 2 and 6 months, but not at 12 months.

To evaluate best cutoff point for PTH to exclude permanent postoperative hypoPT, we performed a ROC curve analysis. The optimal cut-off value for our patient cohort was at 84% decrease in PTH with a sensitivity of 100% (6/6; exact 95% CI 54.0–100%) and a specificity of 85.6% (101/118; exact 95% CI 78.0–91.0%), with an area under the ROC curve of 0.93. At discharge, the optimal cut-off value for our patient cohort was at 77% decrease in PTH with a sensitivity of 1.00, specificity of 0.83, and an area under ROC curve 0.92.

## Discussion

In this study, we have examined the use of ICG fluorescence to predict postoperative hypoPT in a cohort of total thyroidectomy patients. A strong correlation between ICG fluorescence in the parathyroid glands and the change in PTH levels was found. Further, the use of ICG fluorescence was without any adverse events or any adverse effect on liver function. We found a small but significant increase in creatinine levels after TT, with the highest increase for patients developing hypoPT. This finding has been replicated in larger studies[7, 20], however, to the best of our knowledge this is the first time it has been demonstrated just 2 months after surgery. Finally, despite having an ICG score of 2, a few patients did have immediate postoperative hypocalcemia and, subsequently, permanent hypoPT.

Vidal et. al in studies from 2016 and 2018 reported the use of ICG angiography and described an excellent correlation between parathyroid perfusion and function [12, 13]. They found, that one well-vascularized gland could predict normal PTH levels at POD1 and the absence of hypoPT. In the current study, we also found a strong correlation between the need for active vitamin D analogs and ICG measurements. Only 8% of patients with a gland score of 2, needed treatment with active vitamin D at discharge compared with 38% of patients without a gland score of 2 (*p* = 0.001). This correlation however diminished over time, and after 12 months no difference was found between the 2 groups.

From previous studies, it is described, that visual discoloration of the parathyroid gland does not correlate with the health and function of the gland [21, 22]. This problem may be solved using ICG injections to evaluate perfusion. However, some subjectivity is still present when grading a perfusion score between 1 and 2. Lang et. al. has introduced a more objective method of grading the light intensity from the fluorescence by comparing light intensity from the patient’s trachea with the glands using image cursors [23]. Another approach seen in a clinical report explored the use of creating a reproducible and generalizable workflow model of ICG-angiography to reduce subjectivity when grading scores, but needs a larger series [24]. Finally, tissue of interest for fluorescence scoring may have been misclassified as a parathyroid gland, as no histological confirmation was done. Moreover, small tissue in the neck can demonstrate ICG uptake, receive a gland score of 2, and represent false positives. A more objective or quantitative ICG scoring system, and/or the use of autofluorescence, could likely have helped to avoid false-positive glands.

One of the main benefits of ICG fluorescence is the immediate feedback to the surgeon. After dissection of the first lobe, ICG can be injected and the surgeon can control the viability of the parathyroid glands. This helps in intraoperative decision-making in case of no ICG uptake in the two dissected parathyroid glands. This situation leads to a higher risk of postoperative hypoPT and enables the surgeon either to end the operation, to get assistance, or continue on resecting the contralateral lobe with knowledge of the hypoPT risk. It is very similar to the possibility of adjusting the operation after an incidence of palsy on the recurrent laryngeal nerve. Preserving the remaining parathyroid gland tissue may be a more favorable outcome compared to the potential development of hypoPT and the accompanying chronic conditions it brings [7, 8, 25–27]. ICG injections are also used frequently to identify parathyroid arteries and to help preserve these arteries before dissection [28], but that was not part of the current project.

The idea of using the percentage change in PTH is not new. In 2005 Scurry et. al. reported that a less than 75% decrease in PTH and absolute PTH of 7 pg/mL could predict symptomatic hypocalcemia after TT [29]. Similarly, studies report a cut-off value of PTH decrease of less than 70–75% as a safe point for avoiding hypoPT [30, 31]. Our study confirms these findings, as no patients had hypoPT with a decrease below 75% in PTH. Further, the ROC analysis revealed that up to 84% decrease in PTH could be used as cut-off point for permanent hypoPT. This is in contrast to Cavicchi et. al. finding a cutoff value of only 56% PTH drop together with < 7.8 mg/dl calcium 16 h postoperative [32]. We also found a strong correlation between PTH decrease and Ca^2+^ values at POD1. However, after 2 months no correlation was found. Probably due to the treatment of affected patients with calcium supplements and active vitamin D.

We have a large cohort of 124 patients which helps minimize type 2 errors. When determining light emission from the glands after ICG injection, subjectivity can bias the results. To reduce this, all operations were carried out by only 2 surgeons. Prior to the study, they worked through a case series of different perfused glands, to reduce inter-observer variability. Further, when the second ICG dose is injected, there may be a little ICG left from the first injection, which can lead to a score of 2 despite an insufficient vascular supply. Further, since we did not identify all 4 glands in all cases, we did not assess and evaluate all 4 glands in each case. This aspect could clarify why we observed no distinction in PTH or Ca^2+^ values at POD1 between patients with at least one well-perfused gland (score = 2) and those without. Some of these patients may have had a healthy, functioning gland that we did not detect during the evaluation. Similarly, the lack of histological verification of parathyroid glands may cause a misclassification of a score of 2 in other tissue. However, we found it unethical to perform histological verification on all parathyroid glands. Several additional limitations of this study merit acknowledgement. First, the single-centre, nonrandomized design limits the generalizability of our findings. All procedures were performed by only two experienced surgeons, which reduces interobserver variability within the study but may limit applicability to centres with less specialized surgical expertise or where only a single surgeon performs the procedure. Second, a formal a priori power calculation was performed before study initiation; however, the rate of permanent hypoPT was lower than anticipated — reflecting surgical outcomes at our centre that were below national averages — meaning the study was ultimately underpowered for detecting differences in permanent hypoPT between fluorescence groups. Third, follow-up biochemical measurements were offered to all patients at 2, 6, and 12 months; however, a few patients without ongoing hypoPT-related issues declined further blood sampling. Long-term treatment status was reliably ascertained for all patients via the Danish national prescription database, which tracks alfacalcidol prescriptions, mitigating the risk of outcome misclassification. Fourth, the definition of hypoPT was based in part on alfacalcidol therapy initiation; to minimize variability, prescribing followed Danish national guidelines based on PTH and ionized calcium thresholds at POD1, and long-term treatment continuation was independently verified through the national prescription database. Fifth, gland scores were assigned independently by the operating surgeon without inter-surgeon consensus review for uncertain cases. A formal inter-rater reliability was not assessed, which represents a methodological limitation. Finally, the ROC-derived PTH cut-off of 84% for permanent hypoPT is based on only six patients with permanent hypoPT. Although exact binomial confidence intervals have now been calculated (sensitivity 100%, 95% CI 54.0–100%; specificity 85.6%, 95% CI 78.0–91.0%), the small event count limits the robustness of this estimate and external validation in a larger cohort is needed.

## Conclusion

ICG fluorescence provides real-time, reliable feedback on parathyroid gland viability and offers an opportunity for surgeons to make informed decisions during TT, potentially reducing the risk of permanent hypoPT and its associated complications. Further studies with more refined techniques to reduce subjectivity in fluorescence scoring could enhance the applicability and accuracy of ICG fluorescence in clinical practice. The use of ICG in this cohort was not associated with any adverse events, though the sample size is insufficient to exclude rare adverse reactions. No patient developed hypoPT in our cohort with a PTH decrease below 75%.

## Data Availability

All data supporting the findings of this study are available within the paper.
